# Implementation of a Confidential Helpline for Men Having Sex With Men in India

**DOI:** 10.2196/mhealth.3978

**Published:** 2015-02-11

**Authors:** Ashok Agarwal, Myriam Hamdallah, Suvakanta N Swain, Sonali Mukherjee, Neetu Singh, Sudip Mahapatra, Elizabeth J King, Julie Pulerwitz, Ibou Thior

**Affiliations:** ^1^FHI 360New DelhiIndia; ^2^FHI 3601825 Connecticut Avenue, NW, WAUnited States; ^3^UNICEFRanchiIndia; ^4^PATHA-9 Qutab Institutional AreaNew DelhiIndia; ^5^University of Michigan1415 Washington Heights, SPH I, MIUnited States; ^6^Population Council4301 Connecticut AvenueSuite 280NW, WAUnited States; ^7^PATH455 Massachusetts Avenue, NW, WAUnited States

**Keywords:** mobile phone, helpline, MSM, HIV prevention, India

## Abstract

**Background:**

In India, men who have sex with men (MSM) often face physical violence and harassment from police and the general society. Many MSM may not openly disclose their sexual identity, especially if they are married to women and have families. Due to pervasive stigma and discrimination, human immunodeficiency virus (HIV) prevention programs are unable to reach many MSM effectively.

**Objective:**

The objective of this paper was to describe the design, operations, and monitoring of the Sahaay helpline, a mHealth intervention for the MSM population of India.

**Methods:**

We established the “Sahaay” mHealth intervention in 2013; a MSM-dedicated helpline whose main goal was to increase access to comprehensive, community-based HIV prevention services and improve knowledge, attitudes, and behaviors of MSM towards HIV and sexually transmitted infections (STI) in three states of India (Chhattisgarh, Delhi, and Maharashtra). The helpline provided a 24x7 confidential and easy to use interactive voice response system (IVRS) to callers. IVRS function was monitored through an online dashboard of indicators. The system also provided real-time reporting on callers and services provided.

**Results:**

The helpline received more than 100,000 calls from 39,800 callers during the first nine months of operation. The helpline maintained an operational uptime of 99.81% (6450/6462 hours); and answered more than 81.33% (83,050/102,115) of all calls. More than three-fourths of the calls came between 9:00 am-12:00 pm. The most successful promotional activity was “interpersonal communication” (reported by 70.05%, 27,880/39,800, of the callers). Nearly three-fourths of the callers self-identified as MSM, including 17.05% (6786/39,800) as rural MSM and 5.03% (2001/39,800) as a married MSM. Most callers (93.10%, 37,055/39,800) requested information, while some (27.01%, 10,750/39,800) requested counseling on HIV/acquired immune deficiency syndrome (AIDS), STIs, and other health and nonhealth issues. There were 38.97% (15,509/39,800) of the callers that were provided contacts of different HIV/AIDS referral services. Many MSM clients reported increased self-esteem in dealing with their sexual identity and disclosing the same with their family and spouse; and an increase in HIV/AIDS risk-reduction behaviors like consistent condom use and HIV testing.

**Conclusions:**

National HIV/AIDS prevention interventions for MSM in India should consider scaling-up this helpline service across the country. The helpline may serve as an important mechanism for accessing hard-to-reach MSM, and thus improving HIV prevention programing.

## Introduction

### HIV Prevention Programs for Men Who Have Sex With Men in India

Men who have sex with men (MSM) have been substantially affected by HIV epidemics worldwide and better HIV prevention strategies are urgently needed; epidemics in MSM are reemerging in many high-income countries and gaining greater recognition in many low-income and middle-income countries [1]. MSM in India are more likely to be human immunodeficiency virus (HIV) infected and face distinct psychological challenges [2]. The HIV prevalence rate among MSM in India is estimated at 4.4% overall [3], well above that of the general population, 0.27% [4]. HIV/AIDS related stigma is recognized as a major barrier to HIV prevention efforts and an impediment to mitigating its impact on individuals and communities; studies have shown significant associations between HIV-related stigma and low uptake of HIV testing services, unwillingness to disclose test results, and low knowledge about HIV transmission [5,6]. In India MSM are often abused; they face physical violence and harassment from police and the general society. Many MSM may not openly disclose their sexual identity, especially if they are married and have families [7]. Stigma and discrimination acts as an important hindrance for HIV prevention programs targeting different MSM populations. Confronted with significant levels of stigma, discrimination and social exclusion, and limited access to HIV/AIDS prevention and care services, MSM in India are at high risk for HIV infection [8]. The national AIDS control organization (NACO) provides basic targeted intervention (TI) services for MSM, similar to those for other populations at high risk for HIV. These services include counseling and testing for HIV, referral systems for sexually transmitted infection (STI) diagnosis and treatment, availability of free condoms, drop-in centers (safe spaces) at community based organizations or nongovernment organizations (CBOs)/(NGOs), and peer educator outreach [9]. The TI program has been effective, potentially reflected by the trend [10] of declining HIV prevalence among high risk groups. There is emerging evidence that the concerted efforts made in HIV/AIDS prevention and control, particularly among the high risk groups, are having a positive effect on the epidemic, with a 57% reduction in new annual infections over the past decade, the adult general population HIV prevalence declined from 0.41% in the year 2000, to 0.35% in 2006 and to 0.27% in 2011 [11].

However, HIV prevalence has not declined among MSM compared to other high risk population groups [3]. The lack of change may be partly attributable to insufficient information or services accessed, by MSM. In addition, MSM who do not regularly visit cruising sites or drop-in centers are particularly hard-to-reach and have limited access to quality HIV/AIDS prevention services. The NACO recognizes that they have not been able to reach approximately 30% of identified MSM through their interventions [11]. There is an urgent need to reach this population with prevention, care, and treatment messages, as studies [12] have documented their risky sexual behaviors including high numbers of sexual partners, low rates of consistent condom and lubricant use, and high rates of STIs.

### Using Mobile Technology to Promote Behavioral Change

A systematic review of literature on “telephone-delivered interventions” [13] demonstrated the effectiveness of this strategy in promoting behavioral changes such as “increased physical activity” and “dietary changes”. Information and communication technology such as the Internet and mobile phones can deliver behavioral change interventions for STD/HIV prevention and care to more people at low cost [14]. Another review of current research [15] studies published between January 1990 and March 2008 examined mobile telephone short-message service (SMS) for delivering health behavior change interventions via text messages. Positive behavior change outcomes were observed in 13 of the 14 reviewed studies.

An important mHealth experience from the HIV/AIDS sector is that of the “Text Me! Flash Me!” Helpline launched in Ghana in September 2008. It used cell phone technology to provide most-at-risk populations with friendly and accessible HIV/AIDS information, referrals, and counseling services from qualified providers. The evaluation of the project suggested that there was an increase in demand for HIV testing and counseling as well as counseling on STI diagnosis and treatment services. The helpline increased client’s knowledge of and intention to use condom and lubricants [16]. Many countries are also using mobile applications and the Internet for HIV/AIDS intervention [17-19].

mHealth could significantly transform the health care milieu in India by improving health care access for the huge underserved rural population, and enhancing the care for urban residents. India’s high level of mobile phone penetration, with over 911 million subscribers as of February 2012, comprises the world’s  population of mobile phone users. The overall teledensity in India reached 78.1% by the end of February 2012 [20]. The great potentials of mHealth are seen in different settings of India [21].

In 2013, we established the “Sahaay” mHealth Intervention, a MSM-dedicated helpline whose main goal was to increase MSM access to comprehensive, community-based HIV prevention services and improve the HIV/STI related knowledge, attitudes, and behaviors of MSM. The purpose of this paper was to describe the design, operations, and monitoring of the intervention.

## Methods

### Target Population

A consultation with key stakeholders, including representatives from the NACO, MSM community, and various HIV program implementing agencies, was held to outline categories of hard-to-reach MSM, defined as one who is either not registered with a TI project or registered, but has not received any service from a TI in the last six months. The hard-to-reach MSM included: (1) self-identified MSMs, (2) married MSMs, (3) clients of female sex workers, (4) migrant population, (5) clients of male sex workers, (6) rural MSMs, (7) nonself-identified MSMs in cruising sites, (8) male sex workers, (9) MSM people living with HIV/AIDS (PLHIV) network members, and (10) party MSM network members (these MSM are involved in organizing and/or attending sex parties). The categories are not mutually exclusive.

### Study Sites

**Figure 1:**
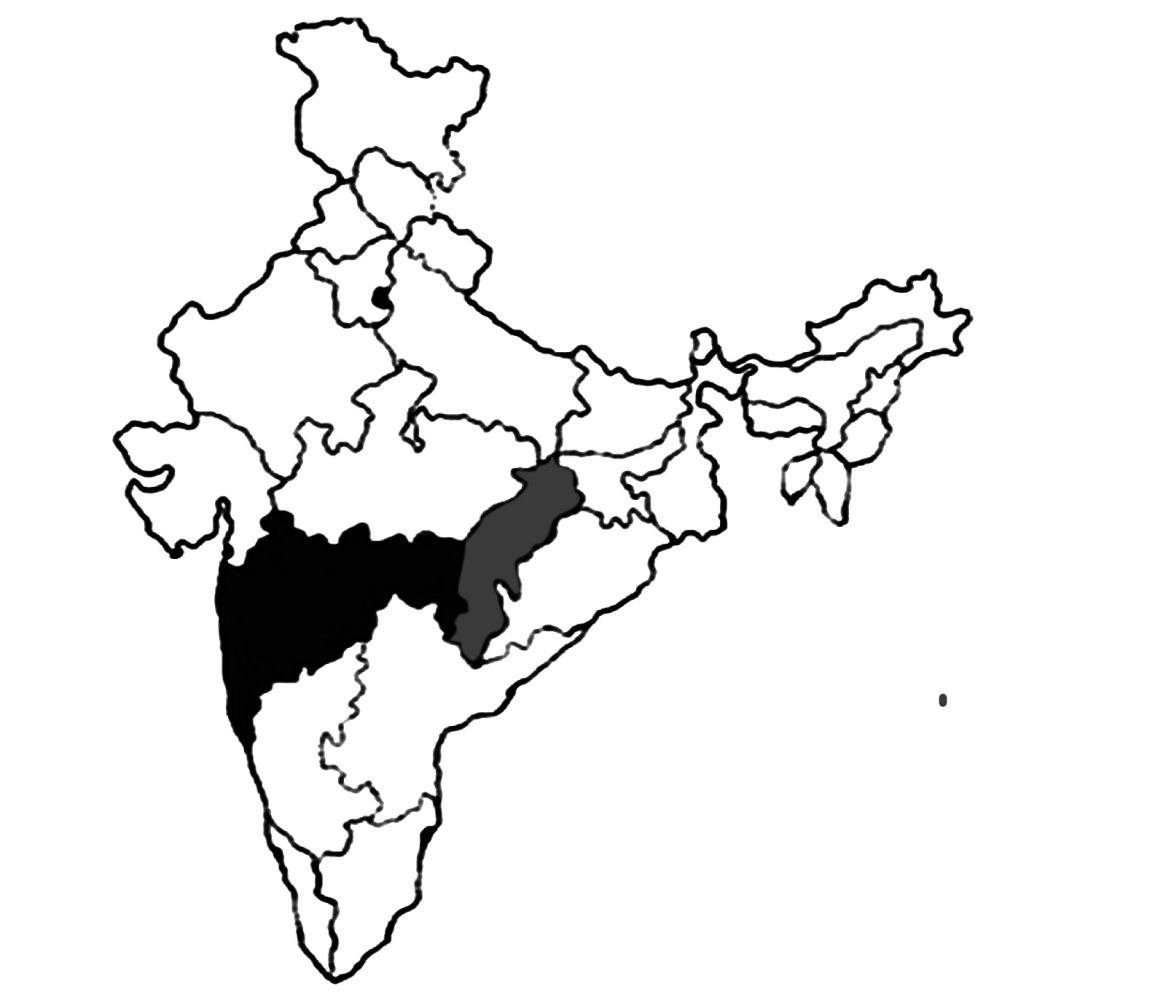
The three study states in India, namely, Chhattisgarh, Delhi, and Maharashtra.

The project included a 9-month intervention in three states of India, namely, Chhattisgarh, Delhi, and Maharashtra, shown in Figure 1.

Family Health International (FHI) 360 worked in coordination with NACO and the State AIDS Control Societies to implement the Sahaay project. The Sahaay helpline operated 24 hours a day, seven days a week, for nine months (September 2013 through May 2014) as the intervention component of the Sahaay mHealth project.

### Intervention

#### Theoretical Basis

The mHealth Sahaay project was based on the Health Belief Model, and the Transtheoretical Model [22]. The Health Belief Model, a psychological model that attempts to explain and predict health behaviors by focusing on the attitudes and beliefs of individuals, was adapted to explore a variety of health behaviors, including sexual risk behaviors and HIV/AIDS transmission. The project used the Transtheoretical Model to provide messages specific to the different stages of condom use (stage 1, not using and no intention to use condoms; stage 2, not using, but has an intention to use condoms in future; stage 3, using condoms sometimes; stage 4, using condoms consistently for less than six months; and stage 5, using condoms consistently for more than six months) of the callers.

#### Helpline Operations

The Sahaay helpline was available to all mobile and fixed phone users. The helpline was managed by four trained counselors in each of the three call centers, serving eight hour shifts (one extra person to account for leaves); at any point of time, there were three counselors available. Counselors with different local dialect backgrounds were purposively hired to provide counseling in local languages as well as Hindi. The duty hours and break-time between the three centers were adjusted to ensure there was no discontinuity in the availability of counselor. Counselors were provided with computer-based software containing a database with a comprehensive list of possible questions and appropriate responses to answer questions promptly and correctly. The database contained an area-wide list of health services that MSM can be referred to for further support and treatment like HIV testing, ant-retroviral therapy (ART), STI diagnosis and treatment, and other general and specialized health issues.

The call centers were housed in three selected MSM-led CBOs that delivered other prevention services for MSM. A caller could receive information and counseling by talking to a counselor, hearing interactive voice response, and receiving text messaging.

**Figure 2:**
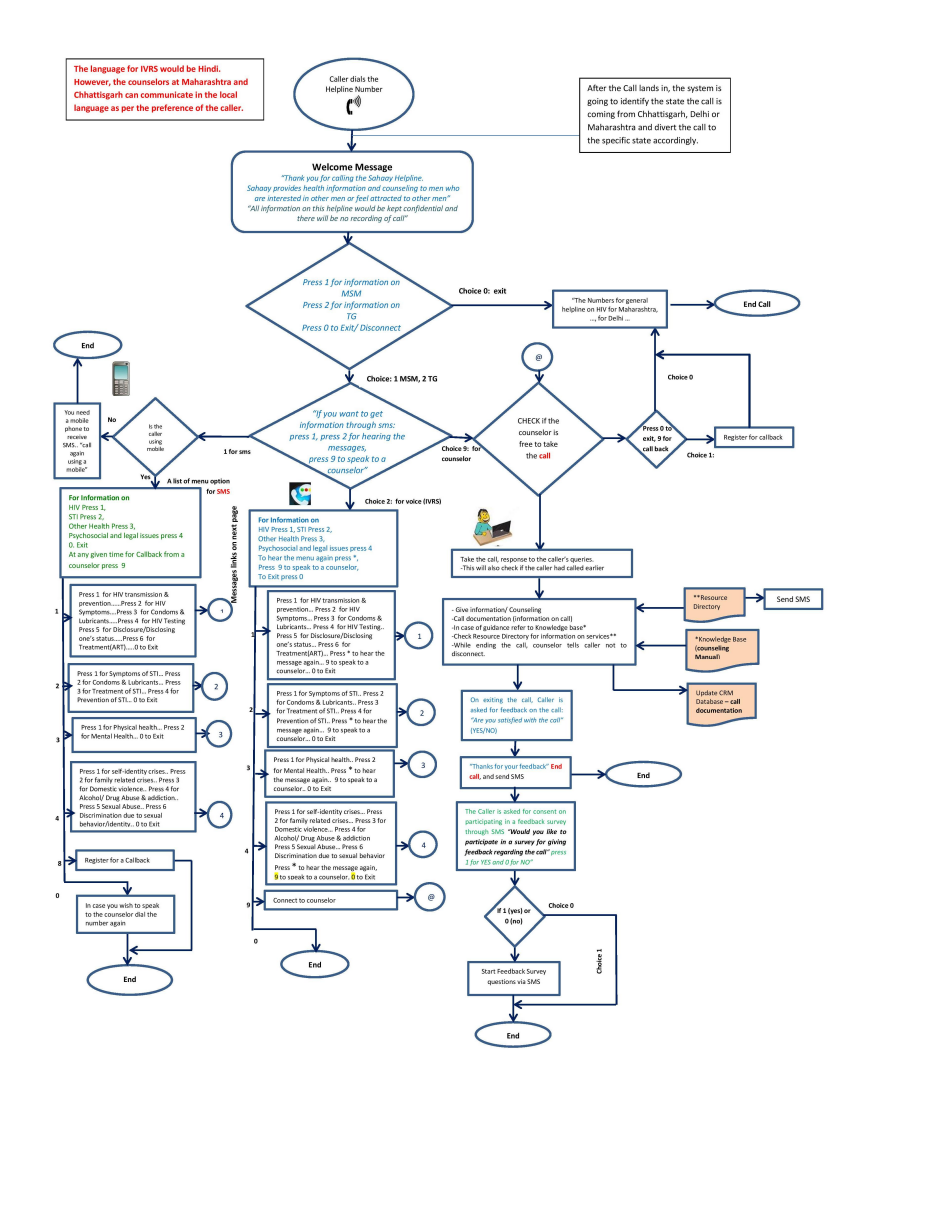
Helpline interactive voice response system (IVRS).

The client interaction process comprised a greeting message, then the helpline assured the caller of the confidentiality, and the interactive voice response system (IVRS) asked the caller if they were seeking information for MSM, transgender (TG), or other groups. For MSM or TG selections, it gave the caller different options for seeking information: talk to a counselor (this option provided the caller with an option of interaction with the counselors and to get information and counseling), voice IVR messages (consisted of prerecorded voice messages which offered options for selecting information), or SMS (when the caller selected SMS as means for information seeking on helpline, the caller was sent a SMS message to select the option from the main list of information) (SMS were charged as per the service provider). Figure 2 shows the detailed IVRS flow chart.

If the selection was not MSM/TG, then the helpline gave the caller the other general helpline numbers for HIV/AIDS (in Delhi and Maharashtra, there was a NACO supported general HIV/AIDS helpline) and the call ended. Calls where clients requested to speak to a counselor were received at any of three call centers in Chhattisgarh, Delhi, and Maharashtra where the counselor was available. In case the caller had a specific language preference, the call was transferred to the location where the counselor spoke the desired language. While the call landed with a counselor, a screen called “Call Documentation Sheet (CDS)” popped up to document the call, with the following indicators: new/duplicate caller, categories of MSM, purpose/issues for calling, accessing other sources of help, awareness of HIV and STI, attitude toward HIV/STI, condom use behavior, HIV testing behavior, willingness to pay for helpline, and how they learned about the helpline. Counselors entered this information on the CDS while speaking to the caller. The CDS also had notes from previous calls if the caller had called previously.

In the event a counselor did not have sufficient information on any topic, s/he had access to a knowledge base in the form of “Counselor Manual” and “Standard Operating Procedure”. In case the caller requested the address of any facility, the counselor had an option of searching through a resource directory and sending the address by SMS to the caller. At the end of the call, the counselor saved the information and the CDS was closed. The counselors also documented select case studies of callers and qualitative data in a notebook. Based on counselors’ feedback, the IVR and SMS messages were updated at the third and sixth month.

#### Services Offered

The following services were provided to the caller: HIV/AIDS counseling, including promotion and referral for HIV testing of callers and their partners to different facilities like TI projects, STI clinics (government run centers for free of cost STI diagnosis and treatment), and Integrated Counseling and Testing Centers (ICTC) (there are 1000s of government HIV testing and counseling centers across the country where the screening and the confirmatory HIV test is done free of cost); and psychosocial counseling, with information and education on HIV/AIDS, MSM risk reduction, and condom and lubricant use.

#### Promotion Activities

##### In the Three Study States

A team of 12 Community Mobilizers (CMs; who had themselves been hard-to-reach MSM earlier), four in each study state, first mapped hot spots patronized by MSM in each district of the state and planned their recruitment approach. CMs then promoted the Sahaay helpline through interpersonal contact in the community, by distributing contact cards at cruising sites, and posting helpline posters and stickers in key locations like government TI projects, MSM preferred health care providers, and beauty and massage parlors where hard-to-reach MSM either work or go for services. The project also developed radio spots that were broadcast through the All India Radio; these particularly reached the rural population.

##### All States of India

Social media, including Facebook, Twitter, and PlanetRomeo, was utilized to disseminate messages widely. Each day a new message was posted on Facebook and Twitter. A promotional video (“BOL NA MERE YAAR”, “Tell me My Friend") posted on YouTube and different other social media channels became very popular. Leading media houses (Times of India, Hindustan Times, Indian Express, and Time Out), prompted by the project’s press releases, published articles on the Internet, and in print.

### Helpline Monitoring

The software program also functioned as a management information system (MIS). It monitored the content of the call and helpline performance. Data generated by the MIS included,

1. Helpline system monitoring data generated automatically and collected daily
2. Counselor call monitoring data, for example, data the counselors documented on the CDS
3. Case studies recorded in the counselor’s notebook; collected during the weekly counselors mentoring done through conference calls by FHI 360 staff.

The FHI 360 staff (Behavior Change Communication Officer and Program Officer) mentored the counselors and the CMs through weekly teleconferences. The CMs were selected from and worked within their respective states (four from each state) to address local outreach activities for hard-to-reach MSM. The CMs reported the number of new hard-to-reach contacted and motivated to make the first call daily through SMS texts.

### Collection of Information and Confidentiality

The caller’s telephone number was not visible to the counselor, but was stored in the server in computer encryption. Clients making multiple calls using the same phone number were shown as duplicate callers on the computer screen of the counselor. The counselor did not ask any personal identifier (like name and address), but discussed HIV/AIDS risk behaviors and provided the required counseling and information on services. The counselor could not open the CDS after saving it. The CDS was stored as an encrypted file in the cloud-based server; consolidated information was generated periodically by the server administrator and used by the project team to monitor the helpline.

## Results

### Target Population

**Figure 3:**
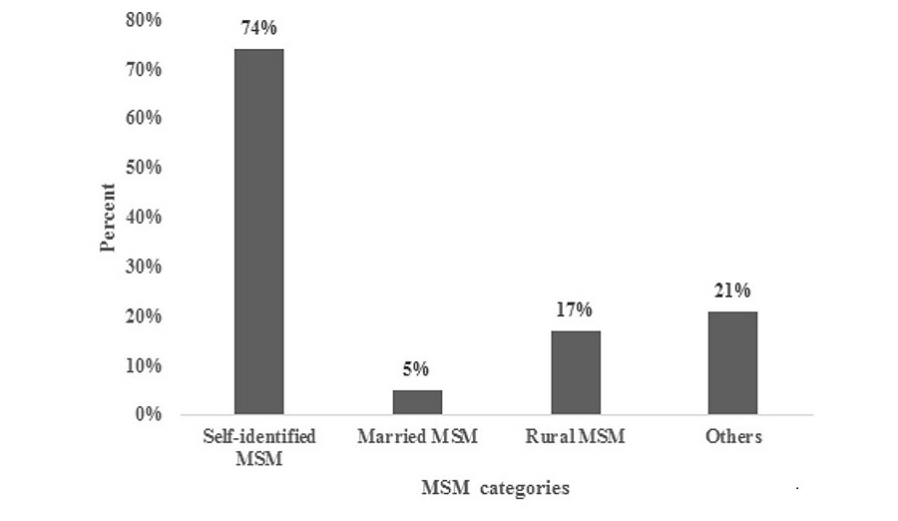
Categories of men who have sex with men (MSM) (N=39,800; multiple counts).

Based on responses given to the counselors, Figure 3 shows the profile of the callers. Nearly three-fourths of the callers self-identified as MSM, including 17.05% (6786/39,800) as rural MSM and 5.03% (2001/39,800) as married MSM. The remaining 21.03% (8368/39,800) were clients of female sex workers, migrants, clients of male sex workers, nonself-identified MSM in cruising sites, male sex workers, or MSM PLHIV network and party MSM network members. Over the operational period, there was an increasing number of calls from MSM belonging to the rural and others categories. During the last quarter of the helpline, only 10.05% (1463/14,559) of callers had accessed services from any CBO, demonstrating that the helpline was gradually accessed by hard-to-reach MSMs who were earlier not reached out by conventional HIV prevention programs.

### Helpline Operations

#### Volume of Calls Received and Key Factors Which Influenced the Volume of Calls

The project had established a daily target of 260 calls to achieve a target of 50,000 unique callers (callers counted as individuals) during the nine months (September 2013 to May 2014) of the helpline intervention. The helpline maintained an operational uptime of 99.81% (6450/6462 hours). Helpline call volume is displayed in Table 1; the total number of callers was 39,800; the number of callers who called more than one time was 46.24% (18,404/39,800). The sizeable percentage of these repeat callers suggests high acceptance of the helpline by MSM. Though the helpline was actively promoted in the three study states (Chhattisgarh, Delhi, and Maharashtra), calls came from all over the country, and a few (0.75%, 766/102,115) came from outside India. The helpline software was unable to document if calls were received from fixed or mobile phones; however, during routine feedback sessions with counselors and CM, they reported anecdotally that callers preferred using mobile phones as this provided them the access on the move and the option of calling from a private location of their choice.

**Table 1:** Sahaay helpline call volume by service request (N=102,115).

Type of calls	Number	Percentage n (%)
Total calls	102,115	102,115 (100.00)
Counselor calls	51,988	51,988 (50.91)
IVR	45,212	45,212 (44.28)
SMS	4915	4915 (4.81)

#### Results of Promotion Activities of Helpline

**Figure 4:**
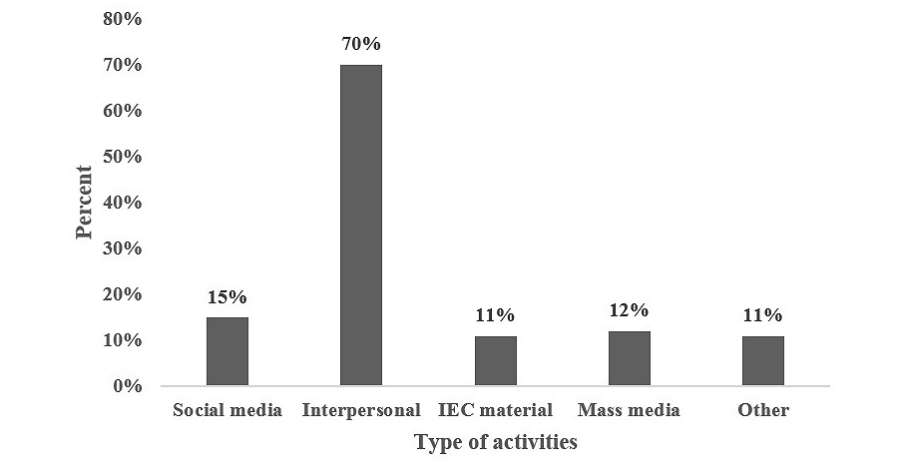
Promotional activities used (N=39,800; multiple counts), (Information, education, and communication = IEC).

Though promotion through social media; information, education, and communication (IEC) materials, news articles, and other modes of communication helped boost the number of calls, the contacts made by CMs indicate a significant role for interpersonal communication in promoting calls from hard-to-reach MSM. As Figure 4 shows, the most successful promotional activity was “interpersonal communication”, which was mostly conducted by CMs in the field, though the data also captured interpersonal communication between stakeholders (different service providers) and MSM; and MSM and their friends and other peers.

Many callers in the beginning did not speak, while others spoke very briefly just to confirm confidentiality, and many callers opened up only after multiple repeat calls. Most callers (93.10%, 37,055/39,800) requested information on HIV/AIDS and STI; some also requested information on issues specific to transgenders, for example, breast enhancement and sex reassignment surgery, while some (27.01%, 10,750/39,800) requested counseling on HIV/AIDS, STIs, and other health and nonhealth issues (many callers requested information and also took counseling; hence the total of information and counseling is >100%; 120.11%, 47,805/39,800). Many callers faced psychosocial problems like “self-identity crisis”, crisis in family relationships, violence and abuse, drug abuse, addiction, or discrimination in the work place or an educational institution. Self-identity crisis included MSM trying either by themselves or forced by others to comprehend their sexual identity (the MSM may not feel attracted to a female and start thinking why this is so with me; or his friends may tease him for his attraction toward a male). Crisis in family relationships included family members pressuring unmarried MSM to get married with women and married men pressured to abandon their sexual life choices. The majority of the callers (79.80%, 31,759/39,800) discussed HIV-related issues, while one-third (34.11%, 13,575/39,800) discussed STI-related issues. A small proportion (15.05%, 5989/39,800) of callers discussed other health issues, including psychosocial problems (4.93%, 295/5989) (many callers discussed multiple issues, hence the total is >100%; 128.95%, 51,323/39,800). Though the project did not measure knowledge of the caller, but they indicated they might know less about STI symptoms and treatment compared to HIV (Table 2).

**Table 2:** Type of information asked on HIV and STI (N=39,800).

Type of information^a^	HIV (31,759; 79.80%) n (%)	STI (13,575; 34.11%) n (%)
Prevention	28,365 (89.31)	9419 (69.38)
Symptoms	16,932 (53.31)	12,856 (94.70)
Condom/lubricants	16,885 (53.17)	8718 (64.22)
Treatment	2958 (9.31)	10,754 (79.22)

^a^multiple counts

#### Referral Services

Many of the callers, (39.25%, 15,622/39,800), asked for and were provided with contacts for various services. Referrals were made to ICTCs (39.02%, 6096/15,622), TI implementing NGOs (CBO/NGO implemented projects working mostly for HIV prevention and referral for HIV testing and treatment services) (22.95%, 3585/15,622), ART centers (government run HIV treatment centers, the services are provided free of cost) (11.04%, 1724/15,622), STI/STD clinics (9.03%, 1411/15,622), Community Care Centers (CBO run centers for providing supporting role in care and treatment of HIV/AIDS, especially during initiation of ART for early detection and management of side-effects of drugs; and where a long-term stay is required for supportive HIV/AIDS care) (6.96%, 1087/15,622), PLHIV MSM networks (5.96%, 931/15,622), and to tuberculosis directly observed treatment center (centers for diagnosing and treating tuberculosis).

#### Condom Use Behavior

Although none of the 39,800 callers reported consistent use of condoms during all sexual acts with all partners for more than six months, they were at various stages of the behavior change continuum, 17.98% (7156/39,800) reported that they did not intend to use condoms; 58.07% (23,111/39,800) would like to use them; 16.03% (6379/39,800) stated that they used condoms sometimes; and 7.96% (3168/39,800) used condoms always, but for less than six months. The counselors explored the reasons for inconsistent condom use. The most common reason provided was “lack of (sexual) satisfaction” (43.28%, 17,224/39,800), followed by unavailability (24.03%, 9565/39,800), partner refusal (15.03%, 5980/39,800), “does not know how to use” (12.01%, 4780/39,800), and “condom breaks” (10.02%, 3988/39,800). Only 1.00% (399/39,800) said, they do not have money to buy or are embarrassed to buy condom.

#### Reasons for Not Testing for HIV

Almost 70% of the callers, or 27,963/39,800, did not report HIV testing in the last one year; the counselor probed for the reasons for not getting tested. The most common reason for not getting tested was “fear” (50.82%, 14,211/27,963) followed by “lack of HIV testing awareness” (23.90%, 6682/27,963), “do not know where to go” (14.74%, 4122/27,963), and “lack of confidentiality at the HIV testing center” (11.12%, 3110/27,963). Only 1.03% (289/27,963) said that they did not have money for testing. Counselors reported that some callers were motivated to get tested after the call, of whom a small group called back to inform the counselors that they had taken an HIV test.

### Monitoring

The Sahaay helpline had a capacity for 32 simultaneous calls including IVR, SMS, and counselor calls. Information technology system support (server, software, and operational glitches) was provided to ensure uninterrupted service around the clock. The helpline was monitored from the FHI 360 India office in Delhi. A continuous monitoring screen facilitated viewing of how many counselors were online and which calls were diverted to which counselor. The FHI 360 team could observe which counselors took the calls and completed the post call documentation. As part of the helpline quality control strategy, the team at the FHI 360 office used “mystery callers” posing as MSM callers with predetermined questions. The mystery calls demonstrated steady improvement in the performance of counselors in addressing the queries of the callers and counseling them on different issues. The helpline answered more than 80% (81.33%, 83,050/102,115) of all calls. More than three-fourths of the calls came between 9:00 am-12:00 pm.

## Discussion

### The Helpline Results Over Nine Months

It is encouraging to note that the helpline received more than 100,000 calls during the nine months of activities reaching out to different profiles of hard-to-reach MSM with information and counseling on HIV/AIDS prevention, care, and treatment. The experience of this helpline is in agreement with the findings of other mHealth service studies, which concluded that these services have wide population reach and can be individually tailored to the caller. This suggests that the service has potential as a delivery channel for HIV prevention and other health behavior interventions. HIV prevention and care programs using digital media have great potential to cost-effectively meet the complex needs of diverse and often underserved populations living with or at high risk of HIV [23]. Delivery of barrier and biomedical interventions with coordinated behavioral and structural strategies could optimize the effectiveness of HIV prevention; modeling suggests that, with sufficient coverage, available interventions are sufficient to avert at least a quarter of new HIV infections in MSM in diverse countries [1]. Unlike some similar interventions in Africa [24,25], which provided one-way SMS and voice message services to recipients, the Sahaay helpline was interactive and easily accessible, even to callers with low literacy, and demonstrates the value of using a confidential, but still human approach via phone counseling.

### Strategies for Behavior Change

Innovative strategies for behavior change communication at individual, group, and community levels may include mobile phone messages, Internet-based strategies, and social marketing campaigns. Current prevention interventions for MSM in India need to consider appropriate modifications to reach out to the hard-to-reach MSM through innovative modalities, like the Sahaay model anonymous helpline service. There are various studies that advocate the use of mobile telephones in scaling up the health care delivery and outcomes. A study by Garai et al [26] argues that intervention studies have demonstrated the applications and effectiveness of mHealth in various health areas in resource poor settings.

Our findings show that interpersonal communication was more helpful in promoting the helpline services as compared to other modes of communication including IEC and social media. As reported from case studies and also from the data on issues requested by callers, there were many calls by those who were suffering from psychosocial issues. External factors, like legal and social barriers often aggravate psychosocial issues facing MSM. India needs HIV prevention approaches that simultaneously address behavioral, biomedical, and structural risks like decriminalization of same-sex behaviors, policies that safeguard MSM and transgender rights, engagement with the media, and community and health systems strengthening with participation of the MSM community. Behavioral approaches to promote safer behaviors to prevent HIV, specifically sustained efforts to increase the use of condoms, should be continued.

### Limitations

Though the Sahaay helpline used a meticulously planned, technically sound monitoring system, the helpline experienced several challenges. Owing to the confidential nature of the helpline, a written referral system could not be implemented. Calls came from throughout the country though the study was located in just three states, and the resource directory of services and facilities developed only included services in the three states, hence the counselors were unable to provide the address of the services and facilities in other states. At times, some prank callers used offensive language; this negatively affected the morale of counselors. Sometimes, there were operational glitches, such as the telephone and Internet line leading to the CDS not opening on the counselor computer, or call disturbances and disconnections. Very few callers accessed the SMS service. The Telecom Regulatory Authority of India did not allow delivery of text messages to mobile phones registered for “do not disturb” facility (in India, one can register with the telephone service provider for preventing calls from marketing agencies). It was also not possible for us to make the SMS service free, as a caller had to dial a number different from the toll free helpline number to access Sahaay messages through SMS. Additionally, the SMS service was available only in English (as most of the commonly used mobile phones in India support English and not Hindi). We also had very low utilization of an automated feedback system, potentially preventing detection and resolution of other technical problems. Because of the Indian Supreme Court ruling criminalizing same sex activity, All India Radio was very selective in terms of allowing messages for broadcast; we had to significantly reduce radio messaging.

### Conclusions

In conclusion, the Sahaay helpline demonstrated that a large number of MSM could be reached over a short period of time from diverse locations in a wide geographic area with a functioning telephonic communication. The helpline was able to penetrate the hard-to-reach MSM groups and promote HIV/AIDS prevention through a nondiscriminatory approach. The helpline is also able to provide information of different health and other services required by the callers. This intervention could be a useful national strategy for reaching out to hard-to-reach MSM in India and other countries. This approach could also be tried for reaching out to other highly stigmatized and marginalized populations.

## Abbreviations

ART: ant-retroviral therapy
CBO: community-based organizations
CDS: Call Documentation Sheet
CM: community mobilizers
FHI: Family Health International
HIV: human immunodeficiency virus
ICTC: Integrated Counseling and Testing Center
IEC: information, education, and communication
IVRS: interactive voice response system
MIS: management information system
MSM: men who have sex with men
NACO: national AIDS control organization
NGOs: nongovernment organizations
PLHIV: people living with HIV/AIDS
SMS: short-message service
STI: sexually transmitted infection
TG: transgender
TI: targeted intervention
